# Management of possible serious bacterial infections in young infants where referral is not possible in the context of existing health system structure in Mbeya, Tanzania: Experience and lessons from the end line assessment

**DOI:** 10.1371/journal.pone.0310259

**Published:** 2024-12-05

**Authors:** Esther Ngadaya, Alexander Manu, Mary Mmweteni, Dorica Burengelo, Doreen Philbert, Gibson Kagaruki, Kahabi Isangula, Mbazi Senkoro, Godfather Kimaro, Amos Kahwa, Fikiri Mazige, Felix Bundala, Nemes Iriya, Francis Donard, Caritas Kitinya, Victor Minja, Festo Nyakairo, Gagan Gupta, Luwei Pearson, Minjoon Kim, Sayoki Mfinanga, Ulrika Baker, Tedbabe Degefie Hailegebriel

**Affiliations:** 1 National Institute for Medical Research-Muhimbili Centre, Dar es Salaam, Tanzania; 2 University of Ghana School of Public Health, Accra, Ghana; 3 London School of Hygiene and Tropical Medicine, London, United Kingdom; 4 UNICEF Tanzania, Dar es Salaam, Tanzania; 5 Aga Khan University, Dar es Salaam, Tanzania; 6 Ministry of Health, Dodoma, Tanzania; 7 WHO Tanzania, Dar es Salaam, Tanzania; 8 UNICEF Headquarters, New York, New York, United States of America; Jimma University, ETHIOPIA

## Abstract

Severe bacterial infections (SBIs) are a leading cause of neonatal deaths in low- and middle-income countries. World Health Organization’s (WHO’s) guideline for outpatient management of danger signs indicating possible serious bacterial infections (PSBI) when referral is not possible was adopted by three pilot district councils in Mbeya Region, in Tanzania (Busekelo, Kyela and Mbarali Districts) in 2018 (the PSBI project). This study documented changes in practice during the PSBI project, and lessons learned. A cross-sectional study was conducted using both qualitative and quantitative data collection methods between July 2021 and January 2022, post-implementation. The study participants comprised stakeholders, health workers, community health workers, and mothers/fathers/caregivers who had a young infant with PSBI. Study tools included record review, quantitative, in-depth, and key informant interviews. Quantitative data were analysed using STATA version 15 (STATACorp Inc., TX, USA), whereas qualitative data were analysed using a framework analysis approach. Our assessment showed that 2,228 young infants (0–59 days old) from the three districts were classified as having PSBI. The majority, 1,607 (72.1%) had fast breathing as the only danger sign, while 621 (27.9%) were classified as having severe illness. All 621 young infants with severe illness were counselled and offered referral to a higher-level health facility; however, only 174 of them (28%) accepted the referral. The remaining 447 severely ill infants, for whom referral was not possible, were treated at the primary health facilities with gentamicin injection and amoxicillin dispersible tablets (DT). When referral is not feasible, outpatient treatment for young infants with signs of PSBI is possible within existing health system in Tanzania, based on experience after this pilot project. However, successful scale-up of outpatient management for PSBI will require commitments from government and key stakeholders to strengthen healthcare systems.

## Introduction

Globally, there is a rapid decline in mortality among children aged under 5 years, but a much slower decline in deaths of newborns, defined as babies in their first month of life [1]. The global under-five mortality rate fell to 38 per 1,000 live births whereas the infant mortality rate (in children aged below 12 months) is 11 per 1,000 live births [2]. The neonatal mortality rate (deaths during the first 28 days of life) accounts for 47% of the global under-five mortality, mostly occurring in low- and middle-income countries (LMICs) [2]. Severe infections are a leading cause of neonatal deaths (>75%), with an estimate of 6.9 million cases of possible serious bacterial infection (PSBI) occurring every year in these countries [3,4].

In the last three decades, Tanzania’s neonatal mortality rate has ranged between 26 and 40 deaths per 1,000 live births, with these deaths contributing more than half of all infant mortality [5]. The major causes of newborn mortality in Tanzania include birth asphyxia (31%), complications of prematurity (25%) and infections (25%). With approximately 43,000 newborn deaths in 2019, Tanzania is among the 10 countries with the highest number of newborn deaths in the world, and among the top five in sub-Saharan Africa [2,5,6].

Moreover, the World Health Organization (WHO) recommendation for managing infections in young infants (0-59 days) is referral for hospital treatment with at least a seven-day course of medication with a combination of two injectable antibiotics – benzyl penicillin or ampicillin plus gentamicin [7]. However, in LMICs, in most cases it is difficult to achieve referral [8]. Furthermore, the quality of care in primary health facilities is often inadequate because of a shortage of trained personnel or basic supplies, which leads to delayed, inadequate or no treatment for many young infants [9]. Simplified regimens of injectable plus oral antibiotics delivered outside the hospital setting, when referral was not possible, have been shown to reduce neonatal mortality due to neonatal sepsis or pneumonia by 25.5% in India [10], 34% in Bangladesh [11] and 12.3% in Pakistan [12]. Similar findings were reported in the Democratic Republic of the Congo, Kenya, and Nigeria. This evidence contributed to the development of the WHO guideline for management of young infants with signs of PSBI when referral is not feasible, published in 2015 [7].

Tanzania, like many other developing countries, still faces a crisis in human resources for health. Physical resources available at all levels of the health system are meagre as well [13–15]. Hence, in 2018/19, the Government of Tanzania, with support from UNICEF and WHO, committed to the adoption, adaptation and national scale-up of management of sick young infants with PSBI in primary level healthcare facilities, starting with one pilot district, Busokelo, and subsequently expanding to two more pilot districts, Kyela and Mbarali, both also in Mbeya Region. The management of PSBI has been incorporated within the integrated management of childhood illness (IMCI) guideline, which is known in Kiswahili as *Matibabu ya magonjwa ya Watoto*, *2019*, on pages 28–29 [16]. The IMCI training package was updated based on the paper-based distance learning course that is being rolled out in Tanzania. The course comprises 15 self-reading modules, and management of PSBI is covered in Module 15, in the section titled “Where referral is not possible”. After three years of implementation, a survey was conducted to assess the management of PSBI in young infants where referral was not possible. This paper describes the experiences and lessons learned after the implementation process in the three-pilot districts in Mbeya, Tanzania.

## Methods

### Study design, setting and period

A cross-sectional study was conducted to assess the management of PSBI in young infants where referral is not possible, between July 2021 and January 2022, employing both quantitative and qualitative data collection methods. The study was conducted in the ‘post-implementation’ phase of the intervention in all three pilot districts in Mbeya Region (Busokelo, Kyela and Mbarali), when the participating health facilities were no longer receiving intensive support from UNICEF and the PSBI project had been handed over to the Tanzania Ministry of Health (MoH). Mbeya Region is one of Tanzania’s 31 administrative regions, located in the country’s southwest, and it has five district councils [17]. There are 22 healthcare facilities in Busokelo, 46 in Kyela and 56 in Mbarali; all were included in the study. In each district there was one district hospital, respectively serving a total population of 109,724 in Busokelo, 221,490 in Kyela and 300,517 in Mbarali.

### Study participants and selection procedure

The study participants included the National Newborn and Child Health Manager from the MoH, as well as district council policy-level stakeholders from the three districts, and representatives from development partners, bilateral and multilateral agencies, including WHO and UNICEF. Other participants were health workers, including health facility in-charges, and mothers/fathers/caregivers who had a young infant with PSBI (Table 1). Moreover, record review was conducted using a record review checklist in all the health facilities to ascertain activities conducted, to assess data quality, and to determine how data were used for essential newborn care during implementation of the PSBI project. The documents reviewed included Health Management information systems including HMIS book 3 and 5, DHIS2 database, CHWs’ registers, and PSBI registers.

**Table 1:** Information requested from the participants.

Participants	Information requested
National Newborn and Child Health Manager from the Tanzania Ministry of Health (MoH) District Council policy-level stakeholders Representative from development partners and bilateral and multilateral agencies, including WHO and UNICEF	Descriptions of their experiences, best practices, lessons learned, and recommendations on how to apply the lessons learned from the pilot implementation at the national level, when the intervention is scaled up, to ensure equity and sustainability
Health facility in-charges	Availability of established facility-based quality improvement (QI) teams, availability of newborn equipment (procured and distributed, and any technical support conducted), availability of gentamicin, amoxicillin, thermometer, weighing scale and timer (observed or reported) and treatment of young infants (aged 0–59) with PSBI
Health workers	Knowledge/skills in managing PSBI in young infants and linkages between healthcare facilities and communities
Mothers/fathers/caregivers	Social, demographic and economic characteristics, knowledge, beliefs and attitude about newborns health, use of services, and healthcare seeking behaviours

#### Sample size calculation

Sample size was calculated to determine the minimum number of caregivers and health workers needed to detect knowledge change around newborn health compared with the baseline level.

The formula below was used to calculate the sample size needed [18–20]:


$$n=D\frac{{\left[Z_{1‐\alpha }\sqrt{2P(1‐P)}+Z_{1‐\beta }\sqrt{P_{1}(1‐P_{1})+P_{2}(1‐P_{2})}\right]}^{2}}{{\left(P_{2}‐P_{1}\right)}^{2}}$$

Where:

D = design effect
P1 = the estimated proportion at the time of the first survey;
P2 = the target proportion at some future date, so that (P2 – P1) is the magnitude of change we postulate to detect at the end of the intervention programme;
P = (P1 + P2)/2;
Z1-α = the z-score corresponding to the desired level of significance;
Z1-β = the z-score corresponding to the desired level of power.

Standard values of Z1-α and Z1-β are provided in statistical probability distribution tables.

All 22 health facilities in Busokelo, 46 in Kyela and 56 in Mbarali district councils were included in the study. The PSBI case management coverage and facility readiness assessments were conducted in all project facilities in the three districts.

**Mothers/fathers/caregivers who had a sick young infant with PSBI:**

To determine the minimum sample size for caregivers, the following statistics were plugged into the formula:

P1 = percentage of women with adequate knowledge of newborn danger signs at baseline, i.e., mentioned at least three danger signs (12.1%); this value was used for this calculation because the baseline data indicated that only 12.1% of interviewed caregivers knew of at least three danger signs/symptoms in newborns.

P2 = percentage of women with adequate knowledge of newborn danger signs at the end line (42.1%), implying 30% absolute magnitude of change targeted by the project at the end line.

Other parameters include: P = (P1 + P2)/2 = 27.1%; design effect (DE) to clear variation between clusters, i.e., districts = 2.0; Statistical Power = 99%; and attrition rate = 20%.

The minimum required sample size (n) for caregivers was thus calculated as 238.

**Health workers:**

To determine the sample size for health workers, the following statistics were plugged into the formula:

P1 = percentage of health workers with adequate knowledge in managing infection in newborns at baseline (7.1%) [21]; this value was taken from the baseline data of a study conducted from Masindi, Uganda, to assess primary health worker knowledge related to prenatal and immediate newborn care, and this value was used since the baseline assessment of the PSBI project did not document this indicator.

P2 = percentage of health workers with adequate knowledge in managing infection in newborns at the end line (30.1%), implying a 23% effect size to be detected at the end line.

Other parameters include: P = (P1 + P2)/2 = 18.6%; design effect (DE) to clear variation between clusters, i.e., districts = 2.0; Power = 95%; and attrition rate = 10%.

The minimum required sample size (n) for health workers was thus calculated as 130.

#### Sampling strategy and sample size

Mothers/fathers/caregivers with young infants (aged 0–59 days) exiting the health facility (after management for PSBI) on the day of the study team’s visit to the facility were invited for exit interviews. In addition, we obtained a list of all young infants aged 0–59 days managed for PSBI who attended the facilities within three months prior to our visit. These caregivers were traced to their communities and invited for interviews. A total of 218 mother/fathers/caregivers were interviewed.

All the health workers who were available and working at the outpatient departments on the day of the study team’s visit were invited to participate in the study. We selected at least one health worker from each health facility who was directly involved in managing PSBI. In total, 133 health workers were included in this assessment giving a response rate of 99.3%. Participants for qualitative interviews were selected based on their involvement in the study, and the total number was determined through reaching saturation point.

### Data collection procedures

The survey instruments were developed using health facility evaluation methodology prepared by the WHO [22] and included: (i) observation checklist; (ii) exit interview with child’s caregiver; (iii) equipment and supply checklist; and (iv) case scenarios (when there was no sick young infant). In addition, guides were developed and used for qualitative interviews. These instruments were pre-tested, and surveyors were trained on their use. The surveyors included experienced research assistants with medical and social science backgrounds. All surveyors could speak good Swahili. Sick young infants aged 0–59 days who presented at any selected health facility during the survey period were enrolled. The survey teams observed the interactions between the caregiver of sick infant and the health worker, which included assessment of PSBI signs, classification of illness, treatment prescribed, referral advice and counselling regarding danger signs and infant care at home. When there was no young infant available on the day of the survey, PSBI case scenarios were used to assess health workers’ ability to diagnose, classify, treat and refer a PSBI case and counsel parents. For the qualitative interviews, both in-depth and KII were conducted in a quiet and isolated room entirely disconnected from regular activities. The audio-taped interview data were gathered using a flexible interview guide to obtain opinions on accountability processes including use of data for decision making on essential new-born care, best practices and lessons learned, and the recommendations on integration of the lessons learned from the implementation into nationwide scale-up. Each KII lasted approximately 60 minutes. More detailed description of the qualitative data collection procedures has been published elsewhere [23]. Briefly, the team also conducted interviews with the caregivers of the sick infants to assess their knowledge of infant danger signs and their perceptions about the services provided at the healthcare facility. The team assessed the healthcare facility’s readiness based on the availability of commodities (medicines and equipment) required to treat PSBI cases. They interviewed the providers about their training/refresher training and supervision status.

### Study variables

The study collected information on three outcome variables: knowledge on newborn danger signs, knowledge on essential new-born care services and overall knowledge percentage scores for all five case scenarios (case scenarios 1,2,3,4 and 5). These were assessed through interviews with mothers/fathers/caregivers and health workers as well as observation during service provision at the facilities.

### Data quality control

NIMR-Muhimbili Centre was directly involved in the protocol development of the research. In collaboration with other members of the core team, they oversaw and monitored the progress of the research implementation and ensured that the proposed quality control measures are accomplished. Challenges encountered were addressed in an ad hoc manner in collaboration with and assistance from UNICEF, and corrective measure were taken accordingly.

### Data management and analysis

#### Quantitative data

Data were collected on both paper-based instruments and electronically using Open Data Kit (ODK). Data from paper-based instruments were entered into ODK and then transferred into Stata version 15 (STATA Corp Inc., TX, USA) for analysis. The data were then rechecked against the paper forms by the survey coordinator. The data were analysed using the priority indicators list developed in consultation with UNICEF. Data were presented using numerical values, charts and tables. We used a t-test and analysis of variance (ANOVA) to statistically assess the mean difference of the continuous variables such as age, knowledge scores, and explanatory categorical variables. We also conducted comparison of binary outcome variables with categorical explanatory variables using a chi-squared test. Association and difference between variables were considered statistically significant if *P*<0.05. To assess an overall knowledge score for management of young infants aged 0–59 days with PSBI among health workers, we used five case scenarios, each with 15 items. A weight of ‘1’ or ‘0’ was given depending on whether the health worker gave a correct or incorrect response, respectively. An individual health worker’s case scores were converted into percentage scores using their total case scores as the numerator and 15 as the denominator. An overall percentage score for all five case scenarios was obtained and was divided by five to get overall average percentage scores per case.

#### Qualitative data

The analysis of qualitative data adopted a ‘framework analysis’ approach for identifying themes related to the research questions. The first stage of analysis was thorough reading through the transcripts to identify these themes and derive codes for subsequent stages of open and then axial coding. These steps broke the qualitative data down into units of analysis, and through this analysis researchers identified patterns and relationships that elucidate the processes identified in the research questions. Explanatory matrices were then developed in relation to each theme, drawing upon the patterns and relationships identified during analysis, and this answered especially PSBI services delivery indicators.

### Ethical approval

Ethical approval was sought from and granted by the National Health Research Review Committee of Tanzania, with ethical clearance number NIMR/HQ/R.8a/Vol. IX/3710. The study team sought permission from all relevant institutions and authorities at the national, regional, district, ward and village levels. Written informed consent was also sought from all individuals prior to their participation in the study. Participation was voluntary and non-involvement had no effect on the participants’ welfare or the care their infants received at the health facility. The interviews were conducted in private, and information obtained remained confidential and was only seen by the personnel involved in the study.

## Results

Data were analysed on knowledge, use of services, and healthcare seeking behaviours of the caregivers of the sick newborn, as well as the quality of care provided to sick young infants (aged 0–59 days) with PSBI from mothers/fathers/caregivers, families, community members and health workers.

One hundred and thirty-three healthcare workers participated in the interviews aimed at ascertaining the quality of care provided to sick young infants using case scenarios and observational checklist. Of the 133 providers, half (n = 67) were males. Their mean age (SD) was 35 years (8.7). Majority (87.2%; n = 116) were from dispensary, owned by the public (88.0%; n = 117). Of the 60 participants who participated in the qualitative interviews, 53% were healthcare workers comprising nurses, clinicians and pharmacists; 22% were healthcare administrators made of DMOs, RCH coordinators and program officers; 17% were CHWs and 8% were mothers whose babies had PSBI.

### Health facility readiness to provide quality management of sick young infants presenting with PSBI

#### Profiles of the health facilities assessed

**Fig 1:**
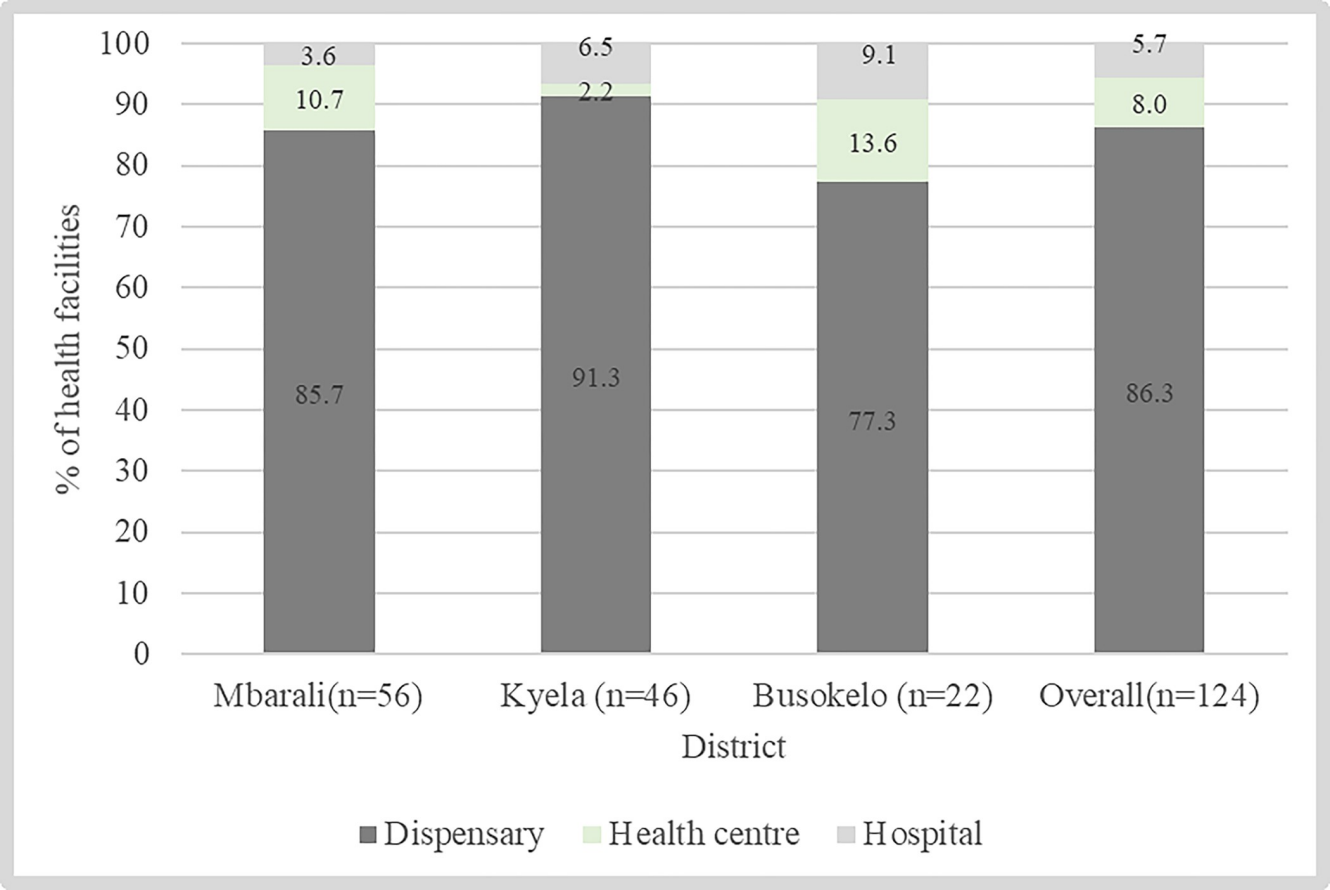
Health facilities by level and district, Mbeya Region.

Out of 124 health facilities, 20.2% (n = 25) were private facilities. Fig 1 presents a list of these health facilities by level, (primary, secondary, tertiary) stratified by district councils.

#### Training of front-line health workers on IMCI/PSBI package in each district

Table 2 below shows the proportion of health workers trained in each district. Out of 368 eligible health workers, 83.7% (n = 308) had been trained on the IMCI/PSBI package.

**Table 2:** Training of health workers on IMCI/PSBI package and QI.

District	Number of health workers eligible for the training	Number (%) of health workers trained on IMCI/PSBI (2018–2020)	Number (%) of health workers trained on quality improvement (QI)	Number of community health workers trained (2019–2020)
Busokelo	75	70 (93.3%)	56 (74.7%)	112
Kyela	143	118 (82.5%)	46 (32.2%)	153
Mbarali	150	120 (80.0%)	74 (49.3%)	150
Total	368	308 (83.7%)	176 (47.8%)	415

Quantitative findings related to training of front-line health workers were supported by qualitative findings. Interviews with participants indicated that the project trained health workers on topics related to categorization and management. The project also trained community health workers (CHWs) and supported them with educational materials and guidelines for provision of health education on infant health to caregivers, and for conducting referral of sick young infants with PSBI to the nearby health facility. Some participants commented:

*“PSBI training focused on diseases affecting the young infants as very severe, severe or not severe, and also treatment. We were also trained on how to use chart books for classification and filling the reports.” (Trained health worker, Mbarali District)*

*“After the training*, *CHWs were given an IMCI booklet*, *posters and some guidelines for use in sensitizing the community*.*” (Health administrator*, *Busokelo District)*

#### Capacity-building to enhance newborn care

Recognizing the importance of ongoing support after the training, the MoH and partners developed the “Guideline for follow-up after IMCI training”. This document provides guidance on key issues that need to be considered during follow-up after any IMCI training with the aim of reinforcing the new skills gained and solving problems encountered in the course of implementing IMCI. Mentorship and follow-up therefore focuses on: case management skills, health facility support, including availability of essential drugs for child health, and documentation and reporting of services offered. During qualitative interviews, participants indicated that the project further supported capacity-building not only with regard to human resources, equipment and supplies, but also with training about on forecasting and quantification of essential medicines including amoxicillin DT and gentamicin injection. Furthermore, joint supportive supervision and mentorship were implemented as part of continued capacity-building after formal training. One participant commented:

*“…The project builds the capacity of district pharmacists and health facility managers on forecasting and quantification of essential medicines including amoxicillin DT and injection gentamicin, particularly during supportive supervision.” (Health administrator, Kyela District)*

#### Quality improvement (QI) for maternal and newborn health (MNH)

Out of 124 health facilities in the three districts, 90.3% (n = 112) had functional QI teams for MNH, defined as QI teams for MNH that meet monthly. The members included medical officer in-charges, clinicians, nurses, pharmacists, health secretaries, health facility administrators, medical attendants, laboratory attendants and data clerks. Main function of this team is to strengthen and promote delivery of quality health services in the health facilities.

#### Availability of essential medicines and equipment

Table 3 shows the proportion of healthcare facilities with gentamicin, amoxicillin, thermometer, weighing scale and respiratory timer available for managing PSBI cases over the three months prior to the assessment visits for this study. In the post-implementation phase of the PSBI project, 82.3% (n = 102) of the facilities had gentamicin injection, 75.0% (n = 93) had amoxicillin DT and over 95% had a functioning thermometer and baby weighing scale. However, only 42.7% of facilities had a functioning respiratory timer, and most health workers reported using the timer on their personal mobile phones for respiratory count. Almost 82% (n = 18) of facilities in Busekelo reported having stocks of amoxicillin DT compared with 76.1% (n = 35) in Kyela and 71.4% (n = 40) in Mbarali. Just under two thirds of the facilities (64.5%, n = 80) had stocks of both gentamicin injection and amoxicillin DT.

**Table 3:** Facilities with gentamicin, amoxicillin, thermometer, weighing scale, and respiratory timer available for PSBI.

Item	Busokelo District (n = 22) N (%)	Kyela District (n = 46) N (%)	Mbarali District (n = 56) N (%)	Overall, (n = 124) N (%)
Functioning respiratory timer	9 (40.8)	20 (43.5)	24 (42.9)	53 (42.7)
Functioning thermometer	22 (100)	41 (89.1)	55 (98.2)	118 (95.2)
Gentamicin injection	13 (59.1	38 (82.6)	51 (91.1)	102 (82.3)
Amoxicillin dispersible tablets (DT)	18 (81.8)	35 (76.1)	40 (71.4)	93 (75.0)
Amoxicillin suspension (250 mg/5ml)	2(9.1)	3 (6.5)	2 (3.6)	7 (5.7)
Amoxicillin 125 mg/5ml suspension	0 (0.0)	0 (0.0)	5 (8.9)	5 (4.0)
Baby weighing scale	22 (100)	45 (97.8)	54 (96.4)	121 (97.6)

### Proportion of young infants with PSBI managed and their treatment outcomes

#### PSBI case identification and outcomes of management at primary healthcare facilities (dispensaries and health centres)

**Fig 2:**
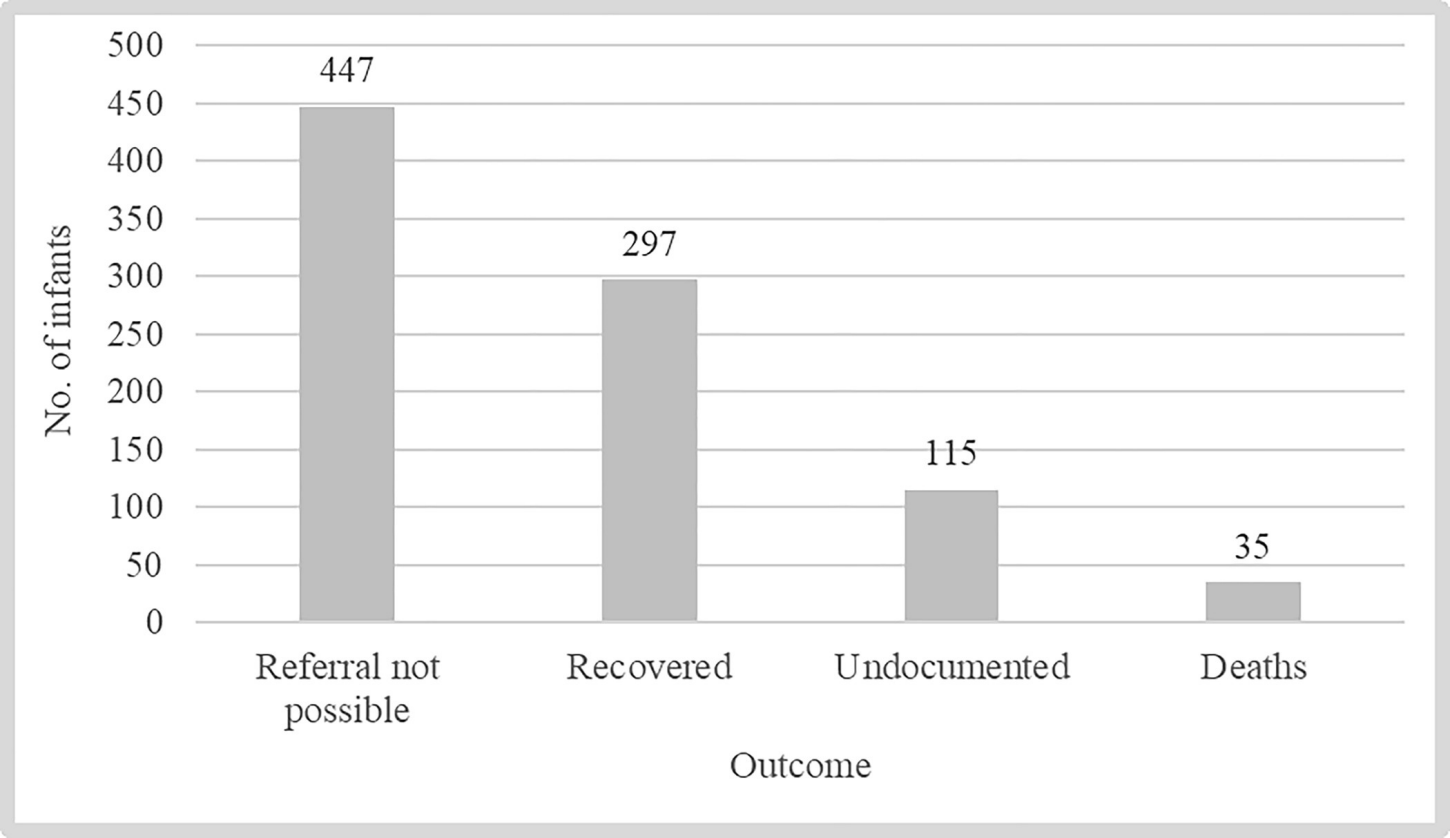
Treatment outcomes of the PSBI cases who received outpatient treatment because referral was not possible (N = 447).

**Fig 3:**
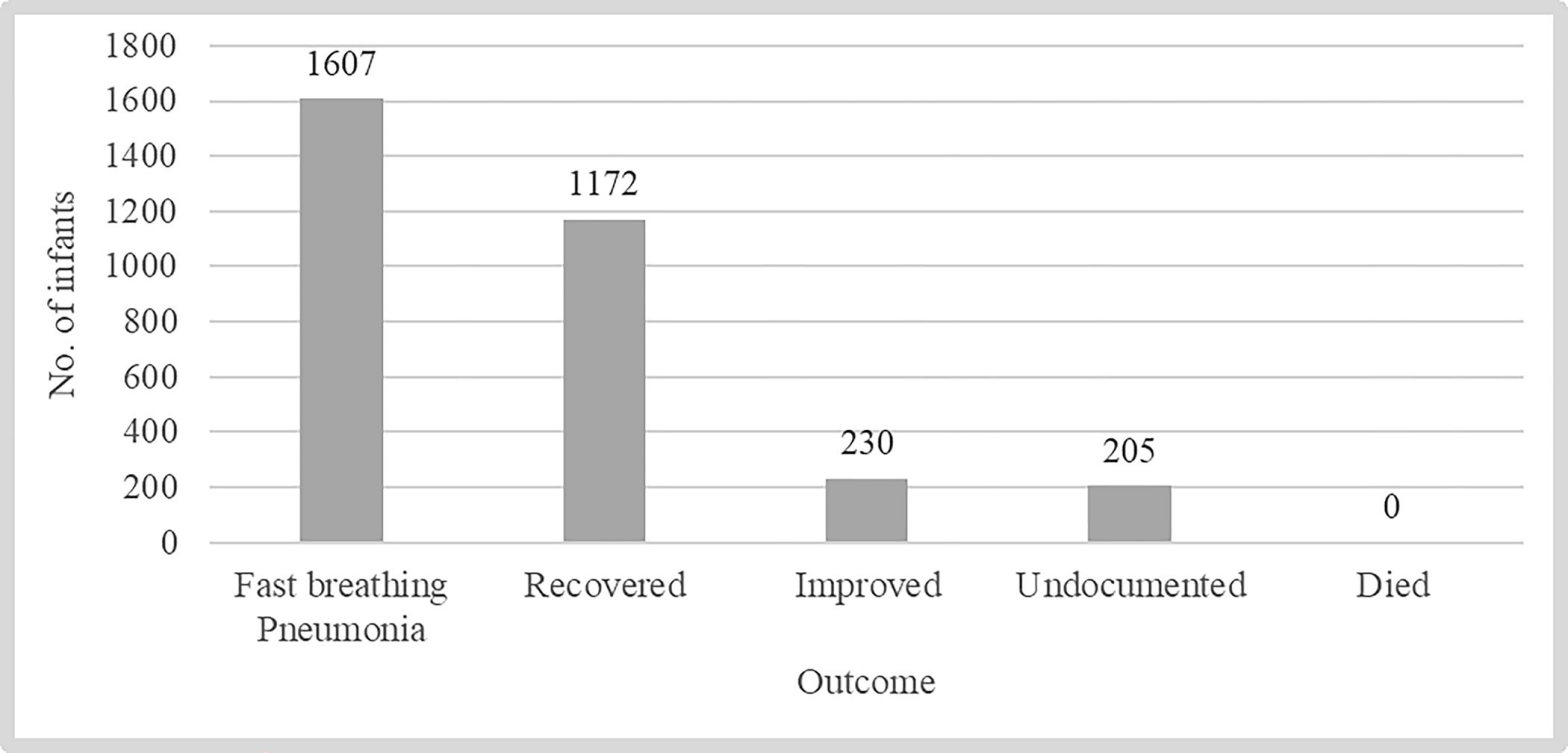
Treatment outcomes of the 1,607 infants who had fast breathing pneumonia and received outpatient treatment.

A total of 2,228 young infants out of a projected 3,295 expected cases (estimated as 10% of all livebirths) from the three districts were classified as having PSBI (Table 4). The majority, 1,607 (72.1%) had fast breathing as the only sign, while 621 (27.9%) were classified as having severe illness, termed as being in the ‘red category’. All 621 young infants in the red category were counselled for referral to a higher-level facility but only 174 (28.0%) accepted the referral. The remaining 447, for whom referral was not possible, were treated at the primary health facility with gentamicin injection and amoxicillin DT, and 184 (41.2%) of them returned for follow-up review on day 4 as requested. As shown in Fig 2, among these 447 cases, 66.4% recovered and 7.8% died, while the remainder were undocumented. Among the 1,607 infants who had fast breathing as the only sign, 1,172 (72.9%) recovered and 230 were reported as ‘improved’, but outcomes for 205 cases were undocumented (Fig 3).

**Table 4:** Number of PSBI cases in young infants estimated from PSBI registers.

District	Number of deliveries during the implementation period (July 2019 – January 2021)	Projected expected PSBI cases (estimated as 10% of live births)	Actual PSBI, from PSBI register	PSBI coverage (%)
Busokelo	8,720	872	739	84.7
Kyela	8,510	851	824	96.8
Mbarali	15,721	1,572	665	42.7
Total	32,951	3,295	2,228	67.6

### Quality of care provided to sick young infants with PSBI at health facilities

#### Health workers’ knowledge on management of infants with PSBI

Health workers’ percent knowledge score in the management of young infants (aged 0–59 days) with PSBI was 80.0%, with 60.2% (n = 80) demonstrating moderate level of knowledge (scored 60.0–79.9%). However, when considering 60.0% as the cut-off point, all the health workers had good knowledge on managing young infants aged 0–59 days with PSBI. Overall, health workers scored the highest in management of a newborn presenting with fever (case scenario 1), with an average mean percentage score of over 90% across all districts. More than two thirds (70.0%) of the providers had good knowledge on management of a child presenting with fast breathing (case scenario 4) (Table 5). There was no significant difference in knowledge on the management of young infants presenting with different signs of PSBI across all sociodemographic characteristics (Table 6).

**Table 5:** Average scores on different case scenarios by districts.

Case scenario	Case	Kyela District Mean % (SD)	Mbarali District Mean % (SD)	Busokelo District Mean % (SD)
1	Fever	96.5 (7.9)	93.9 (9.9)	94.8 (9.7)
2	Fast breathing pneumonia	79.6 (12.3)	73.9 (14.0)	75.5 (14.3)
3	No symptoms	91.6 (7.8)	89.3 (9.3)	87.0 (12.2)
4	Fast breathing pneumonia	70.6 (11.3)	68.9 (9.8)	70.9 (10.2)
5	Lethargy	71.2 (13.3)	70.1 (13.3)	72.7 (13.0)
1-5	Overall	81.9 (5.9)	79.2 (5.9)	80.2 (5.8)

**Table 6:** Overall average correct knowledge percentage scores by social demographic characteristics.

Variable	Case 1, Mean (sd)	Case 2, Mean (sd)	Case 3, Mean (sd)	Case 4, Mean (sd)	Case 5, Mean (sd)	All 1-5 cases Mean(sd)
District						
Kyela	96.5(7.9)	79.6(12.3)	91.6(7.8)	70.6(11.3)	71.2(14.5)	81.9(5.7)
Mbarali	93.9(9.9)	73.9(14.0)	89.3(9.3)	68.9(9.8)	70.1(13.3)	79.2(5.9)
Busokelo	94.8(9.7)	75.5(14.3)	87.0(12.3)*	70.9(10.2)	72.7(13.0)	80.2(5.8)
Sex						
Male	95.3(8.1)	76.0(14.3)	88.7(9.6)	69.5(10.0)	70.5(13.9)	80.0(5.8)
Female	94.6(10.3)	76.3(13.1)	90.8(9.2)	70.2(10.8)	71.4(13.5)	80.7(6.0)
Age group						
<30yrs	95.4(8.9)	77.9(14.1)	92.3(6.7)	67.1(9.4)	69.0(13.5)	80.3(5.4)
30-60yrs	94.8(9.3)	75.6(13.5)	89.0(10.0)*	70.7(10.5)	71.5(13.7)	80.3(6.0)
Working experience period group						
<5yrs	93.4(10.7)	76.8(14.0)	90.9(8.2)	67.9(9.8)	69.4(13.4)	79.7(5.6)
5+yrs	95.6(8.5)	75.9(13.6)	89.2(9.9)	70.6(10.5)	71.6(13.8)	80.6(6.0)
Working experience period group in the same facility						
<5yrs	94.3(9.1)	75.8(14.1)	89.7(9.6)	69.2(10.4)	70.3(13.5)	79.9(6.1)
5+yrs	95.8(9.4)	76.6(13.2)	89.8(9.3)	70.6(10.4)	71.7(13.9)	80.9(5.7)
Working experience period group current post						
≤3yrs	94.0(9.5)	75.2(14.6)	89.2(9.7)	68.7(10.7)	71.8(13.4)	79.8(6.3)
>3yrs	95.8(9.0)	77.0(12.8)	90.2(9.2)	70.8(10.0)	70.1(13.9)	80.8(5.5)
Health facility level						
Dispensary	95.0(9.2)	76.3(13.5)	89.7(9.2)	69.6(10.7)	70.9(13.8)	79.9(5.9)
Health centre	96.4(8.3)	77.2(14.6)	91.1(9.6)	72.2(7.4)	72.2(14.2)	82.7(5.6)
Hospital	90.0(10.8)	69.3(16.7)	86.7(15.6)	69.3(7.6)	68.0(11.0)	83.3(5.0)
Ownership						
Public	95.4(9.2)	77.5(13.2)**	90.0(9.4)	70.3(10.5)	71.6(13.8)	80.2(6.0)
Private (FBO)	92.0(9.0)	66.3(13.0)	87.9(10.1)	66.7(8.8)	65.8(11.9)	80.8(5.6)
Education level						
Primary/secondary	95.6(8.2)	80.0(12.1)	93.3(5.6)	70.7(8.6)	73.0(16.2)	82.5(5.1)
College	94.8(9.3)	75.5(13.8)	89.2(9.8)**	69.7(10.6)	70.6(13.2)	80.0(6.0)
Marital status						
Married	94.4(9.8)	75.3(13.5)	89.5(9.8)	71.1(10.6)	71.5(13.6)	80.4(6.0)
Not married	96.3(7.3)	78.2(13.9)	90.3(8.7)	66.5(9.1)	69.4(13.7)	80.1(5.8)

^*‡*^*P-value<0*.*001*

**<0.01

*<0.05.

While quantitative surveys focused on establishing the quality of care at the health facility by determining the knowledge and skills of health workers using case studies and an observational checklist, qualitative interviews examined perspectives of health workers, health administrators and mothers of infants with PSBI to further assess the quality of care. Mothers were included because the project had committed to use periodic feedback from mothers and communities. Qualitative analysis of the transcripts indicated that there was a broad consensus among participants that the care provided for sick infants at the facility was of good quality. Participants used four dimensions to judge the quality of care: (i) availability of guidelines and essential medicines, (ii) expedited care, (iii) good interpersonal relationships with providers and (iv) mentorship/supportive supervision and follow-up.

Participants suggested that UNICEF ensured not only availability but also use of PSBI and IMCI guidelines for treatment of young children and provided ongoing mentorship through supportive supervision and follow-up to facilitate health workers’ adherence to PSBI guidelines instead of relying on common knowledge. The project was said to have ensured availability of essential medicines at all the health facilities for management of sick newborns. Monthly and quarterly supportive supervision sessions were conducted and there was frequent close follow-up of services’ provided, to ensure correct management of sick newborns at the facilities and proper documentation. Likewise, mothers of infants with PSBI were happy with the expedited care and friendliness of health workers. The assertions of friendliness of health workers were also identified as a key driver of mothers’ satisfaction with care, me participants commented:

*“UNICEF facilitated all facilities to have PSBI and IMCI guidelines and they were given wall posters and flow charts which highlight on danger signs and steps for management. In terms of medications, we ensured availability of amoxycillin as well as gentamicin injections for newborns and other medications needed for PSBI in all facilities. As CHMT (Council Health Management Team), we were supported financially to conduct mentorship every month. We followed up management offered at the facilities. Follow-up was received well and we had different follow-up activities monthly and quarterly.” (Health administrator, Busokelo District)*

“We ensured quality of care through frequent mentorship that we conducted monthly to ensure that all services provided follow the guideline, that the services are of good standard and based on the guideline instead of personal predictions. Therefore, we visited facilities as a district team, instructed them on how to offer better services to the client.” (Health administrator, Mbarali District)

*“The services are good*. *We do not sit for a very long time*… *we only use around half an hour only*. *The provider is not harsh and they offer good care*.*” (Mother of an infant with PSBI*, *Busokelo District)*

### Knowledge, use of services and healthcare seeking behaviours among caregivers of sick newborns

Of the 218 caregivers, 89.0% (n = 194) were the mothers of the sick young infants. Almost two thirds had primary education. Caregivers’ mean age was 26.9 (SD: 7.7) years. Over 80% (n = 179) were either married or cohabiting. Overall, 67.4% (n = 147) reported the father to be the main decision-maker in the family. Over half (58.3%; n = 127) of the heads of the households and 63% (n = 138) of mothers were engaged in subsistence farming. Over half (54.6%; n = 119) reported that it took over 30 minutes to reach the nearest health facility. At the end line assessment, there was a significantly higher proportion of caregivers with the knowledge of at least three newborn danger signs 69.7% (n = 152), compared with 12.2% (n = 33) at baseline (*P*<0.001) (Table 7). The most common danger signs cited included high temperature (78.0%), feeding poorly or not feeding at all (36.2%), convulsions (36.2%) and fast breathing (30.7%).

**Table 7:** Proportion of mothers/fathers/caregivers with knowledge of at least three danger signs, comparing baseline and end line assessment.

Phase	Number	Percentage (95% CI)
Baseline	33	12.2% (4.4–29.4%)
End line	218	69.7% (63.3–75.5%) ***
Change		57.5% (44.8–70.2%)

***P-value<0.001.

Almost all the young infants (215 out of 218; 98.6%) were delivered in a health facility, with no difference across the districts surveyed: Busokelo (98.3%), Mbarali (98.8%) and Kyela (98.7%) (Chi-square = 0.078 and *P* = 0.962). Overall, 84.9% (n = 185) of the caregivers with sick young infants sought medical care from an appropriate health facility (primary care level or higher) within 24 hours of appearance of danger signs. Fever was the most common sign for which caregivers brought their infants to health facilities for care (89.5%). More than 80% (n = 172) of the caregivers reported that the care received was good, and 98.2% (n = 214) were satisfied with the healthcare services provided. Furthermore, 97.3% (n = 212) would attend the same health facility they had visited the next time their children fell ill. Nearly two thirds (65.1%;l n = 142) of the recently delivered women reported being counselled on danger signs in newborns after delivery.

To complement quantitative findings related to healthcare seeking, qualitative interviews were done with mothers/fathers/caregivers and health workers that sought to examine the drivers of the preference for formal healthcare services for sick young infants over informal or traditional practices. One of the drivers of this preference was proximity to the health facility. The findings indicate that the shorter the distance to the healthcare facility, the greater the likelihood of seeking hospital care for sick newborns, and the inverse is also true – the further the distance, the lower the likelihood. Another driver was the young age of a sick child. Some participants suggested that the younger the age of the sick newborn, the higher the likelihood of seeking hospital care. Moreover, the availability of many formal health workers in the area (public and private health facilities and laboratories) emerged as another driver. Additionally, community sensitization through education by health workers (and CHWs) on the importance of seeking care at health facility was another driver. Although some caregivers indicated a possible preference for informal care, when they were asked about traditional healers, some participants discredited such practices. On the one hand, those who stated preferring traditional healers over health facilities a view that “traditional practices cannot be eradicated completely”, as one health worker from Busokelo pointed out. On the other hand, those discrediting informal/traditional practices or practitioners gave as their main reason their religious-backed trust towards Western medical care and fear of traditional practices, cemented by the historical presence of Christian missionaries in the locality. Religious beliefs were also cited as a driver for seeking prayer services among some parents of sick newborns before visiting health facilities if they felt they may have been the target of bewitchment. Preference for healthcare facilities over traditional healers was also associated with residence in urban areas. Some participants commented:

*“[When a child is sick] I personally take her to the hospital because I do not live far from the facility. Some goes to drug outlets when the child is sick because they live far from the facility.” (Mother of an infant with PSBI, Kyela District).*

“Most families in this community prefer seeking care from healthcare facilities. Very few seek care from traditional healers before coming to health facilities but most come to facilities because we have offered them education and sensitization therefore, they don’t see the need to go to traditional healers.” (Health worker, Busekelo District)

“They often come to hospitals. In the surrounding communities no one is going to traditional healers because historically this area is dominated by missionaries, therefore people have religious upbringing which at least creates fear towards traditional healers… maybe if they go outside this village. However, …it is also common to find them visiting surrounding drug outlets before visiting hospitals but not for young children…no… we have not seen that… it is only common for older children… We also have community health workers who for example when they encounter a woman who accidentally gave birth at home, they accompany her and the newborn to the hospital. Also, a person who harbours feelings of bewitchment or bad spirits may first visit a religious leader for prayers and if unsuccessful they come to hospital although such kind of people are very few and I never personally met one.” (Health worker, Kyela District)

*“We seek care from drug outlets and hospitals*. *Regarding traditional healers*, *our village is in urban area therefore people do not go to them*. *Those who go are mainly pregnant women nearing delivery seeking medicines to reduce labour pain and speed up delivery*.*” (Mother of an infant with PSBI*, *Mbarali District)*

## Discussion and analysis of results

In a cohort of 32,951 infants born alive between January 2019 to July 2021 in the districts of Busekelo, Kyela and Mbarali, 2,228 (6.8%) young infants aged 0–59 days met symptomatic criteria for PSBI. The incidence of PSBI in the first six weeks of life observed in this study is similar to the 7.6% global PSBI incidence reported in 2012 [24]. Our coverage of identifying and treating PSBI cases was 68%, similar to the 70% observed in India [25]. Among those sick young infants brought to the healthcare facilities, almost three quarters had fast breathing as the only danger sign, while just over a quarter (621; 27.9%) were classified as having severe illness. All caregivers of the 621 severely ill young infants were counselled for referral to a higher-level health facility but only 28.0% accepted the referral. The referral acceptance rate observed in this study is similar to that reported in India [26]; slightly over two thirds of the young infants were not referred but were treated at a primary health facility with gentamicin injection and amoxicillin DT, of whom 7.8% died. The PSBI case fatality rate (CFR) observed in this study is slightly higher than that observed in Kenya, but consistent with that reported in Pakistan [27], and slightly lower than the CFR of 9.8% described in a systematic review/meta-analysis from low- and middle-income countries [24]. If these young infants had not been offered this simplified antibiotic therapy when their families refused referral, it is quite likely that many more would have died. Previously published research has shown that prompt and appropriate treatment can reduce the CFR in young infants with severe infection by 30–70% [28,29], making it one of the most important strategies for improving newborn survival. Implementation of outpatient treatment when referral is not possible is critical for low-income countries where rates of referral refusal are high [30–33]. The low CFR may be attributed to factors including high knowledge of danger signs among caregivers and health workers, early referrals to healthcare facilities and quality of care improvements observed in this study. This study also reported very high facility-based delivery (98%), and over 6 in 10 women had been counselled on danger signs in newborns before discharge following delivery. Moreover, the prevalence of essential neonatal care practices was higher than has been reported elsewhere [34,35], which may also have contributed to the low CFR. About 40% of all under-five deaths worldwide have been attributed to suboptimal newborn care practices [36].

In the implementation of PSBI management, healthcare facilities, including private facilities in the three pilot districts, became better at managing sick young infants with PSBI. Incorporating PSBI management within the IMCI guideline, involvement of key stakeholders dealing with improving newborn health in the country – such as the Paediatric Association of Tanzania (PAT) and WHO – and registration and importation of paediatric formulations of gentamicin injection 20 mg/1 ml and amoxicillin DT 250 mg in the country were among the important stepping stones and key lessons learned from this study.

Community–government partnerships for integrated health improvements established by the MoH with support from UNICEF, WHO and other professional organizations, is the ideal model for disseminating best practices and lessons learned, as well as overcoming the barriers to increasing the acceptability/accessibility/utilization of PSBI interventions. The emphasis on private–public partnership to strengthen health service capacity and accountability added complexity but reaped rewards in terms of collaboration and ownership. However, efforts are required to maintain the visibility of the positive findings obtained in Mbeya Region and to scale up the PSBI interventions throughout the country.

The project facilitated capacity-building of front-line health workers on management of essential supplies and commodities (IMCI/PSBI Module 14), such that capacity was built among district pharmacists and in-charges of the health facilities for forecasting and quantification of essential medicines including amoxicillin DT and gentamicin injection. Additionally, all 124 facilities were supplied with drugs, equipment and supplies for the management of young infants aged 0–59 days with PSBI.

Our findings also showed that multiple modalities were used in the implementation of the PSBI project in the three districts, through existing partnerships between the MoH, UNICEF, PAT and WHO. Initial meetings and workshops to review guidelines and policies, updating the IMCI training package [23], clinical site assessment together with the district council health management teams and UNICEF not only provided stakeholders with the opportunity to give technical support to the programme but was also a necessity for the programme buy-in. Furthermore, the project was designed to operate at the interface between the district- and national-level government authorities, involving close technical support for the implementation research to ensure smooth implementation and to oversee the developments on the ground. The establishment of such an institutional platform at the district level with technical support from the regional and national level and/or from UNICEF, means that PAT and WHO can play a facilitator role when new interventions are rolled out or when implementation challenges arise within existing programmes. Studies elsewhere have reported the importance of stakeholders in providing organization-specific adaptions to evidence-based interventions, to ensure effective adoption, implementation and sustainability in the local context [37–41].

Capacity-building under the PSBI pilot project in Tanzania was implemented taking into consideration the societal perspective that where capacity-building is applicable to both health workers and community members. The capacity-building initiatives as well as the provision of equipment and supplies enhanced management of young infants with PSBI and thus improved newborn care. The project managed to train almost 90% of the eligible health workers. Health worker training, supportive supervision visits and mentorship during project implementation improved knowledge and skills on management of young infants with PSBI. Overall, the mean percentage knowledge scores for managing young infants (0–59 days of age) with PSBI among health workers was 80%, with 60.2% (n = 80) demonstrating moderate level of knowledge (score range: 60.0–79.9%). Lack of knowledge in managing young infants with PSBI in India was associated with lack of confidence and being apprehensive about administering gentamicin injections to young infants [25,26,42–44].

Community advocacy messages circulated included newborn danger signs, illness recognition, care seeking and male involvement in newborn care – all together, these improved the proportion of caregivers who know at least three dangers signs from 12.5% at the baseline to 69.7% at the end line. This study also documented both high rates of hospital deliveries (98.6%) and high rates of seeking care promptly (within 24 hours of the appearance of danger signs) from an appropriate health facility (84.9%).

Another important lesson learned, as revealed during this study, was the importance of quality improvement (QI) activities for maternal and newborn health (MNH), when implemented together with the PSBI project. These activities included establishing functional QI teams in over 90% of the healthcare facilities and development of QI plans. In addition to QI of MNH services, improved data management, reporting and data use to enhance the quality of care provided were also established. QI may be one of the reasons why caregivers were satisfied with care received from the healthcare facilities, and almost all (97.3%) would attend the same facility they had visited the next time their children fell ill. QI in healthcare services delivery was reported to be a valuable opportunity for individuals to be involved in leading and delivering change, from improving individual PSBI patient care to transforming services across complex health and care systems [45].

## Conclusion

We demonstrated that effective management of young infants aged 0–59 days with PSBI is feasible at primary healthcare facilities in a resource-constrained country like Tanzania, when referral to a higher-level facility is not possible. However, stakeholders’ involvement, review of guidelines and policies, capacity-building both at healthcare facilities and in communities, providing equipment and supplies, and monitoring and supportive supervision should all be in place well in advance of implementation. Sufficient resources also need to be allocated to improve CHW’s involvement in the management of young infants with PSBI.

## Supporting information

S1 Dataset *Mothers and caretakers data set. *Healthcare workers data set. *Health facility tool data set.
(ZIP)
